# Youth Mental Health First Aid Training: Impact on the Ability to Recognize and Support Youth Needs

**DOI:** 10.1007/s11414-024-09893-4

**Published:** 2024-08-01

**Authors:** Sara Geierstanger, Jessica Yu, Melissa Saphir, Samira Soleimanpour

**Affiliations:** 1 Philip R. Lee Institute for Health Policy Studies, University of California, San Francisco, San Francisco, CA USA; 2 Philip R. Lee Institute for Health Policy Studies and Department of Epidemiology and Biostatistics, University of California, San Francisco, San Francisco, CA USA

## Abstract

Youth Mental Health First Aid (YMHFA) trains individuals who regularly interact with youth to identify youth experiencing mental health challenges. Several studies demonstrate positive training impacts, but few assess whether the training equally impacts participants of different demographic and professional backgrounds or those who participate in different training modalities. Using a pre-post follow-up design with a comparison group, this study examined changes in participants’ confidence in their ability to recognize and support youth mental health needs 1 to 2 months after training. Data were collected over two years (2021–2023) from training participants (*n* = 480) and comparable non-participants (*n* = 51). The authors examined whether changes in confidence varied by participant race/ethnicity, professional role in the education or mental health fields, and training modality (online versus hybrid). Training participants’ confidence in supporting youth mental health increased significantly compared to non-participants. Although the training was effective for all participants, those with less mental health experience benefited more, consistent with previous research. While both in-person and hybrid training were effective, in-person training participants reported slightly higher confidence scores than virtual at follow-up. Study findings suggest that educational and social service organizations should offer this training to their staff and community members who interact with youth, prioritizing participants with less prior mental health training and delivering training through an in-person training modality when possible. However, additional research is needed to explore how aspects of in-person training, such as trainer characteristics and group dynamics, impact outcomes.

## Introduction

Youth Mental Health First Aid (YMHFA) is a training that aims to teach adults who regularly interact with youth, including parents/caregivers, school staff, health and human services workers, and other caring adults, how to help adolescents (ages 12–18 years) who are experiencing mental health challenges or are in crisis. YMHFA trainings are conducted virtually, in person, or through both formats. The trainings introduce participants to adolescent development and common mental health challenges and teaches a five-step action plan to help young people in crisis and non-crisis situations. Two recent systematic reviews of research evaluating the efficacy of YMHFA training found consistent positive outcomes among training participants, including increased mental health knowledge and confidence in recognizing and addressing mental health concerns.^1,2^ To ensure that as many adolescents as possible receive effective mental health support, researchers evaluating YMHFA outcomes are now focusing on the impact on different types of adult participants.

There is widespread agreement that, in the short term, YMHFA training benefits professionals from educational and social service workforce groups and parents/guardians. For example, one study conducted with social service providers found a significant increase immediately after training in the likelihood of providing help and identifying appropriate responses to youth mental health challenges.^3^ Another study with school staff reported a significant increase in confidence immediately after training.^4^ Furthermore, YMHFA training was found to have increased skills for front-line workers in juvenile justice settings,^5^ as well as increased knowledge, confidence, preparedness, and intentions to intervene across participants from various occupational fields, including child welfare, education, support services, and the justice system.^6^ Finally, training was also found to be effective among parents/guardians, including increasing mental health knowledge, attitudes toward help-seeking, and intentions to help.^7^

Several studies have also examined YMHFA’s longer-term impacts and found that increases in awareness about youth mental health and confidence in aiding youth with mental health challenges persist over time. An early study from Australia found that confidence and knowledge gains following the YMHFA training persisted at 6-month follow-up among adults from the general community.^8^ A more recent study found that positive effects of the YMHFA training, including increased confidence, persisted at 3-month follow-up among non-mental-health workers.^9^ Another study observed improvements at 6-month follow-up compared to pre-training in knowledge, attitudes, and beliefs and in motivation to help youth address their mental health problems.^10^

As might be expected, given that those with less prior training have more room to improve, several studies suggest that the YMHFA is more effective among those with less prior mental health education. Larger improvements in mental health literacy and confidence were observed among participants who were not mental health providers compared to those who were providers.^9^ Having below-average confidence and knowledge about youth mental health needs at pre-training was associated with larger and sustained improvements at follow-up compared to above-average participants.^11^ Participants with no prior mental health education had higher gains in outcomes, such as self-confidence and positivity, than those with previous training.^4,10^ In contrast to these findings, another study found that prior training did not significantly predict trainees’ confidence or preparedness in addressing mental health issues after accounting for demographic characteristics and satisfaction with the course.^6^ A study of social work students concluded that the YMHFA training was effective for students both with and without prior mental health education but did not compare outcomes between the two groups.^12^ Further studies are needed to clarify whether YMHFA training is equally effective for participants with different levels and types of prior mental health education.

Research on the efficacy of the training among participants of different racial and ethnic backgrounds also lacks consensus. For example, one study found that, compared to white participants, those who identified as other racial or ethnic backgrounds increased more from pretest to posttest in mental health literacy.^4^ In contrast, another study found that race did not explain significant variance in post-training outcomes.^13^ Two adaptations of the YMHFA training have been shown to be effective among diverse participants. However, these studies had small sample sizes and did not compare one racial or ethnic group to another. An Australian study concluded that culturally and linguistically diverse YMHFA training improved outcomes among an ethnically diverse group of participants.^14^ Recent research has also demonstrated the effectiveness of adapting the YMHFA training for Asian American participants in terms of improved mental health literacy, knowledge, and confidence.^15,16^

During the COVID-19 pandemic, YMHFA training began to be offered virtually, given that in-person training could not be held. As pandemic restrictions eased and in-person activities resumed, virtual training continued, given the logistical advantages for instructors and participants. The authors are unaware of research examining the effect of training modality (e.g., in-person versus virtual) on training outcomes. The present study addresses this gap as well as questions that remain from the research reviewed above about the effects of participant characteristics on training outcomes. Specifically, this study assesses the impact of YMHFA training on participants’ confidence in recognizing, communicating with, and supporting youth in emotional distress. It examines whether these outcomes vary by participants’ race/ethnicity, educator and mental health professional roles, and training modality.

## Methods

### YMHFA curriculum

The YMHFA training was developed in Australia in 2007 as a 12- to 14-h course to teach educators and other community members who interact with adolescents how to help youth with mental health problems.^17–19^ First implemented in the USA in 2008, the National Council for Mental Wellbeing adapted the course to a 6- to 8-h training. The course teaches participants how to assess a mental health crisis that a youth is experiencing, select the appropriate response, and connect the youth to support and self-help resources. The training content describes adolescent development, signs and symptoms of mental health challenges, how to implement a five-step action plan to help young people in both crisis and non-crisis situations, and self-care for the YMHFA responder. The five-step action plan is referred to as “ALGEE,” which stands for: *Assess* for risk of suicide or harm; *Listen* nonjudgmentally; *Give* reassurance and information; *Encourage* appropriate professional help; and *Encourage* self-help and other support strategies.

### Instructor training and participant outreach

The California Department of Education (CDE) trains and certifies local instructors to teach the YMHFA course, conducts outreach to school communities, and offers the training at no cost to their school staff and other adults who interact with youth. They also coordinate the scheduling of the YMHFA training, conduct the registration of participants, and offer a stipend to instructors, including travel reimbursement for in-person training.

### Participants

The CDE collaborated with an external university-based team to conduct an evaluation study of the YMHFA training. The evaluation team collected data from individuals throughout California who registered for the YMHFA training through the CDE between July 2021 and June 2023. The evaluation was reviewed and approved by the University’s Institutional Review Board. During the 2-year study period, YMHFA instructors taught 168 training sessions in 32 counties throughout California. The average number of participants in each training was 14. For those who participated in the YMHFA course virtually, 2 h of self-paced online pre-work were required, followed by 6.5 h of online training led by instructors. The in-person content was identical to the virtual modality but covered during 6.5 h of in-person instruction. Both the virtual and in-person training were conducted in English.

During the study period, 909 individuals registered for the training, of whom 780 completed the entire training, 21 only partially completed the training, and 108 did not attend the training at all. All 909 individuals who registered for the training were emailed a request to complete an online seven-question follow-up survey 4 to 6 weeks after the training. The study sample was limited to those who participated in the entire training and whose time between finishing the training and completing the survey was between 27 and 73 days (the intervention group) and those who registered but did not complete the training (the comparison group). Participants who partially completed the training were excluded because only two had completed the pre-training survey. The sample was restricted to include only those who took both a pre- and post-survey and had complete data on study measures related to confidence (246 participants who did not meet these criteria were excluded). The final sample who met these criteria comprised 429 individuals who completed the training (intervention group) and 51 individuals who registered but did not complete the training at all (comparison group).

### Measures

The baseline measures for this study were collected via the CDE registration form for the YMHFA course. The follow-up measures are from the survey sent to all registrants. Registrants for the YMHFA training reported their race/ethnicity, educational role, previous mental health education, and whether they were mental health providers in the CDE registration form. Because the race/ethnicity question was added to the registration form in spring 2022, approximately one-quarter of the study participants (*n* = 125 in the intervention group and *n* = 13 in the comparison group) needed to include data for this variable. The education role variable was dichotomous, with registrants who marked any of the 24 educational roles (e.g., school counselor, teacher) coded 1, and those who selected “Does not apply, I do not work in education” coded 0. For mental health education, registrants reported whether they had “no previous mental health training,” attended “a few talks and presentations,” attended “several workshops or classes,” or had a “graduate degree and license in mental health.” Finally, a mental health provider was defined as a school or community-based mental health provider (e.g., marriage and family therapist, licensed clinical social worker, associate in social work, licensed professional clinical counselor, school psychologist, school social worker, school counselor, school nurse, all coded 1). All others were categorized as non-mental health providers (coded 0).

Seven questions on the registration form and follow-up survey measured participants’ confidence in recognizing and supporting youth experiencing a mental health crisis (Table 1). These questions were based on the actions that YMHFA is intended to increase and were used in prior YMHFA evaluation studies.^9,10^ Response options were “Not at all confident” (coded 1), “A little confident (2), “Moderately confident” (3), and “Extremely confident” (4). Participants were asked about their confidence in areas of “ALGEE” action plan, including being able to (1) recognize when a child/youth’s behavior is a sign of emotional distress; (2) assess youth for risk of suicide or self-harm; (3) listen non-judgmentally when a youth talks about their feelings or experiences; (4) give reassurance that help is available to a child/youth experiencing emotional distress; (5) encourage a youth experiencing emotional distress to get appropriate professional help (e.g., talk to a counselor or doctor, or call a crisis hotline); (6) encourage a youth experiencing emotional distress to use self-help strategies (e.g., books, websites, meditation); and (7) refer youth for mental health support services. Finally, based on information provided by the training instructors, the YMHFA trainings were coded as virtual or in-person.

**Table 1:** Baseline characteristics of intervention and comparison groups

Characteristics	Intervention (*n* = 429) % (*n*)	Comparison (*n* = 51) % (*n*)	*p* value
Race/ethnicity			0.30
White	22 (95)	10 (5)	
Latino	32 (135)	39 (20)	
Black	4 (18)	8 (4)	
Asian/Pacific Islander	6 (27)	8 (4)	
Multiple race/ethnicities or “other”	7 (29)	10 (5)	
Missing	29 (125)	25 (13)	
Educator role			0.20
Yes	79 (341)	71 (36)	
No	21 (88)	29 (15)	
Mental health provider			0.38
Yes	26 (111)	23 (5)	
No	69 (294)	67 (31)	
Missing	5 (24)	10 (5)	
Prior mental health education			0.73
No previous mental health training	16 (67)	16 (8)	
A few talks and presentations	33 (143)	27 (14)	
Several workshops or classes	28 (120)	33 (17)	
Graduate degree/license in mental health	15 (64)	12 (6)	
Missing	8 (34)	12 (6)	
Training modality			
Virtual	90 (386)	See note^a^	
In-person	10 (43)		

^a^Training modality was relevant only for the intervention group

### Analyses

Chi-square tests were used to determine whether the intervention and comparison groups had significantly different demographic characteristics at baseline. To determine whether there were significant improvements from baseline to follow-up, paired *t*-tests were conducted to compare confidence scores for each of the seven confidence outcomes at baseline and follow-up. For each study participant, the authors subtracted the average confidence score on the pre-test from the average score on the post-test to obtain a measure of change in confidence. Two-sample *t*-tests and ANOVA with Bonferroni corrections were conducted to compare change scores across demographic groups within the intervention group. Lastly, a multivariate ordinary least squares (OLS) regression model was used to identify which demographic characteristics, previous mental health experience, and training modality were significant predictors of change scores. Diagnostic tests showed that the assumptions for OLS regression were met, and errors were normally distributed. All statistical analyses were performed using RStudio (R 4.0.2), and a significance level of *p* < 0.05 was used.

Given the sample size imbalance between the intervention (*n* = 429) and comparison groups (*n* = 51), the authors conducted sensitivity analysis employing inverse probability weighting (IPW). IPW involves calculating weights based on propensity scores, which represent the probability of being assigned to the intervention or comparison group given the observed covariates.^20^ Applying these weights to the data before running the *t*-tests and ANOVAs balances the covariate distributions between the groups, effectively creating a pseudo-population in which the groups are more comparable.

## Results

### Study participants

There were no significant differences between the intervention and comparison groups (Table 1) regarding demographic characteristics, professional roles, and prior mental health education. Among both groups, over 30% were Latino, 21% were white, and 29% did not provide their ethnicity. Over two-thirds of both groups worked as educators (79% of the intervention group, 71% comparison) and were not mental health providers (69% of the intervention group, 67% comparison). Those in educator roles were mainly teachers (30% of the intervention group, 28% of the comparison group), paraprofessional/instructional aides (13% intervention, 14% comparison), and school counselors or social workers (18% intervention, 28% comparison). Non-educators were mostly employers of youth (47% intervention, 67% comparison) and parent/guardians (40% intervention, 22% comparison). Nearly half of the intervention and comparison groups described themselves as trained in mental health, having attended “several workshops, classes” or having a “graduate degree and/or license” in mental health. Of the intervention group, 90% attended the course virtually, and 10% participated in-person.

### Changes at 1–2-month follow-up in confidence (intervention and comparison groups)

Overall, the intervention group exhibited greater gains in each confidence outcome measure from baseline to follow-up than the comparison group, which did not significantly improve at follow-up (Table 2). Among the intervention group, confidence scores improved significantly for every racial/ethnic group, mental health providers and non-providers, educators and non-educators, and those with and without prior mental health education. The intervention participants increased by 0.734 points on the four-point confidence scale (from 2.942 to 3.676; *p* < 0.001), compared to a non-significant increase of 0.1508 among the comparison group (from 2.93 3.02 to 3.0810). The weighted sensitivity analysis controlling for the imbalanced sizes of the treatment and comparison groups produced substantively similar results.

**Table 2:** Pre-post scores for intervention and comparison group

	Intervention			Comparison		
Outcome	Pre Mean (SD)	Post Mean (SD)	*p* value	Pre Mean (SD)	Post Mean (SD)	*p* value
Recognize distress	2.88 (0.7)	3.59 (0.5)	< 0.001	3.04 (0.7)	3.00 (0.7)	0.77
Assess risk	2.50 (0.9)	3.46 (0.6)	< 0.001	2.59 (0.8)	2.63 (0.9)	0.82
Listen	3.38 (0.7)	3.83 (0.4)	< 0.001	3.61 (0.6)	3.53 (0.6)	0.50
Reassure	3.08 (0.8)	3.76 (0.5)	< 0.001	3.18 (0.7)	3.32 (0.7)	0.32
Encourage professional help	3.05 (0.8)	3.74 (0.5)	< 0.001	3.18 (0.8)	3.20 (0.8)	0.90
Encourage self help	2.84 (0.9)	3.63 (0.6)	< 0.001	2.98 (0.7)	3.16 (0.8)	0.25
Refer to services	2.71 (0.9)	3.58 (0.6)	< 0.001	2.59 (0.9)	2.90 (0.9)	0.09
**Average confidence score**	**2.92 (0.7)**	**3.66 (0.4)**	** < 0.001**	**3.02 (0.5)**	**3.10 (0.6)**	**0.47**

### Differences in confidence change scores by participant characteristics (intervention group only)

The bivariate analyses within the intervention group revealed significant differences in the size of confidence change scores by three participant characteristics (Table 3). Confidence change scores were higher for non-mental health providers than mental health providers, for those with less previous mental health training compared to those with more prior training, and for those who completed the training in-person versus virtually. In other words, among those who completed the YMHFA training, confidence increased more for participants who were not mental health providers, had less prior training, or completed the training in person compared to their counterparts in the intervention group. Significant differences in confidence change scores were not found between racial/ethnic groups or between those who worked in education roles and those who did not.

**Table 3:** Differences in confidence change scores by participant characteristics among intervention group (*n* = 304)^c^

Characteristics	Mean change score (SD)	*p* value
Race/ethnicity		
White	0.72 (0.6)	See note^a^
Latino	0.76 (0.6)	
Black	0.60 (0.6)	
Asian/Pacific Islander	0.86 (0.7)	
Multiple race/ethnicities or “Other”	0.57 (0.5)	
Educator role		
Yes	0.72 (0.6)	0.41
No	0.79 (0.7)	
Mental health provider		
Yes	0.56 (0.5)	< 0.01
No	0.81 (0.6)	
Prior mental health education		
No previous mental health training	1.07 (0.8)	See note^b^
A few talks and presentations	0.80 (0.6)	
Several workshops or classes	0.67 (0.5)	
Graduate degree/license	0.33 (0.4)	
Training modality		
Virtual	0.70 (0.6)	< 0.01
In-person	1.02 (0.8)	

^a^Pairwise comparisons between all groups were not significant

^b^Pairwise comparisons between all groups were significant at *p* < 0.01, except for the difference in mean scores for those who had attended “a few talks and presentations” and those who had attended “several workshops and classes,” which were not significant (*p* = 0.43)

^c^125 intervention group participants were excluded due to missing race/ethnicity information

### Predictors of confidence change score (intervention group only)

When all the measured participant characteristics were entered into a regression model predicting confidence change scores among those who completed the training, race/ethnicity, being a mental health provider, and being an educator were not significant predictors of improvement in confidence (Table 4). Prior mental health training and training modality were the only significant predictors of confidence change scores. Compared to those who had a graduate degree or license in mental health, participants who had no previous mental health training increased in confidence by 0.64 points more on the four-point confidence scale after completing training, holding all other variables constant (*β* =  − 0.64, *t*(293) =  − 5.12, *p* < 0.001). Participants who completed the training in person increased their confidence by 0.40 points more than those who completed the training virtually (*β* =  − 0.40, *t*(293) =  − 2.65, *p* < 0.001).

**Table 4:** Regression model predicting change score among intervention group (*n* = 304)^a^

Predictors	Coefficient (*β*)	SE
Educator role	0.01	0.08
Mental health provider	− 0.07	0.09
Prior mental health education		
A few talks and presentations	− 0.22***	0.10
Several workshops or classes	− 0.37***	0.10
Graduate degree/license in mental health	− 0.64***	0.13
Race (reference group = white)		
Asian	0.06	0.12
Black	− 0.14	0.15
Latino	− 0.01	0.08
Multiple race/ethnicities or “other”	− 0.16	0.12
Training modality		
Virtual	− 0.40***	0.15

^a^125 intervention group participants were excluded from the OLS regression model due to missing race/ethnicity information

^***^p < 0.001

## Discussion

This study assessed the effectiveness of YMHFA training on participants’ confidence in recognizing, communicating with, and supporting youth in emotional distress and examines how these outcomes vary by participant characteristics and training modality. The current study corroborated prior research findings that the YMHFA training significantly increases participants’ confidence in recognizing and supporting youth experiencing a mental health crisis.^1,2^ Individuals who completed the YMHFA training showed significantly greater gains in each measure of confidence from baseline to follow-up 1 or 2 months later than those who registered for but did not complete the training. This is an important finding because few prior studies have evaluated outcomes for YMHFA training participants versus a comparison group, particularly those in educational fields.^2^

Among participants who completed YMHFA training, confidence scores improved significantly from baseline to follow-up for each racial/ethnic group, and there were no significant differences in changes among them. These findings contribute to the limited research on demographic differences of YMHFA training impacts and demonstrate that, at least in this California sample, the training is equally effective for all racial/ethnic groups. While research supports the positive effects of adaptations for specific ethnic or cultural groups,^15,16^ these findings suggest that the current curriculum content can be effective for participants of diverse racial/ethnic backgrounds.

Among those who completed YMHFA training, confidence improved equally for educators and non-educators. In bivariate analyses, improvement in confidence was greater for non-mental health providers than for mental health providers, consistent with previous study findings.^6^ However, being a mental health provider did not predict improvement in confidence after controlling for other participant characteristics. Prior mental health training was the only participant characteristic that remained a significant predictor of change after controlling for other characteristics. Consistent with previous research,^4,9,11^ the current study found that the YMHFA training was most effective for participants with the least mental health training. Although the YMHFA training was shown to be effective for all individuals regardless of prior mental health training, individuals who have less prior mental health training benefitted the most.

To the authors’ knowledge, this is the first published evaluation of YMHFA training conducted during and immediately following the most severe period of the COVID-19 pandemic, which closed most California public schools entirely to in-person instruction during the 2021–2022 school year. At that time, nearly all professional development training switched to an online format, and most YMHFA trainings were conducted virtually during the 2022–2023 school year. There were significant improvements in confidence among participants who took the course virtually and those who took the course in person. However, confidence increased significantly more for those who completed the training in person than those taking the classes virtually. This could mean that, while virtual implementation is inexpensive and effective, participants may gain more from in-person interactions and settings. Additional research is needed to explore other factors influencing these outcome differences, such as trainer characteristics and group dynamics.

### Limitations

One limitation of this study is that it did not assess differences in the quality of instruction across instructors. Although all instructors had the required YMHFA teaching certification, additional characteristics of individual trainers might account for some differences between the virtual and in-person training formats. A second limitation is that this study did not measure additional training that participants may have taken between completing the YMHFA training and the follow-up survey up to 2 months later, which could account for some differences observed between the intervention and comparison groups. Finally, this study did not randomly assign individuals to intervention and control groups. Thus, some of the differences observed between the two groups may be attributable to unmeasured characteristics that motivated individuals in the intervention group to complete the training.

## Implications for Behavioral Health and Future Research

Study findings hold several implications for practice. First, evidence of the effectiveness of YMHFA training provides further justification for educational and social service organizations to offer this training to their staff and community members interacting with youth. Second, although participants with all levels of mental health training reported significant improvements in confidence in recognizing, communicating with, and supporting youth in emotional distress, these results suggest that to optimize training benefits and to use resources more efficiently, organizations offering the training might consider prioritizing the recruitment of participants with less prior mental health training. Next, although both in-person and virtual training modalities significantly improved participant confidence levels, the improvements were slightly greater among those who received in-person training. This finding suggests that providing the training in person would be most beneficial when possible. However, virtual training has many advantages, particularly for communities in more rural settings.

Additional research is needed to identify which aspects of in-person training contribute to differential effects, as the knowledge gained could be used to improve both training modalities. A simple way to start would be to collect participants’ perceptions of the strengths and weaknesses of the virtual and in-person versions of the YMHFA training. Future research should also examine the need for and impact of offering the training in languages other than English, as well as incorporating more extended follow-up periods to determine how long training effects last.

## Data Availability

All authors confirm that all data, coding, and materials support their published claims and comply with field standards.
